# The Leukemic Stem Cell Niche: Adaptation to “Hypoxia” versus Oncogene Addiction

**DOI:** 10.1155/2017/4979474

**Published:** 2017-06-04

**Authors:** Giulia Cheloni, Martina Poteti, Silvia Bono, Erico Masala, Nathalie M. Mazure, Elisabetta Rovida, Matteo Lulli, Persio Dello Sbarba

**Affiliations:** ^1^Department of Experimental and Clinical Biomedical Sciences, Università degli Studi di Firenze, Florence, Italy; ^2^Istituto Toscano Tumori, Florence, Italy; ^3^Department of Medical Biotechnologies (Ph.D. Programme), Università degli Studi di Siena, Siena, Italy; ^4^Department of Experimental and Clinical Medicine, Università degli Studi di Firenze, Florence, Italy; ^5^Institute for Research on Cancer and Ageing of Nice (IRCAN), UMR CNRS 7284-INSERM U1081, Université de Nice Sophia-Antipolis, Nice, France

## Abstract

Previous studies based on low oxygen concentrations in the incubation atmosphere revealed that metabolic factors govern the maintenance of normal hematopoietic or leukemic stem cells (HSC and LSC). The physiological oxygen concentration in tissues ranges between 0.1 and 5.0%. Stem cell niches (SCN) are placed in tissue areas at the lower end of this range (“hypoxic” SCN), to which stem cells are metabolically adapted and where they are selectively hosted. The data reported here indicated that driver oncogenic proteins of several leukemias are suppressed following cell incubation at oxygen concentration compatible with SCN physiology. This suppression is likely to represent a key positive regulator of LSC survival and maintenance (self-renewal) within the SCN. On the other hand, LSC committed to differentiation, unable to stand suppression because of addiction to oncogenic signalling, would be unfit to home in SCN. The loss of oncogene addiction in SCN-adapted LSC has a consequence of crucial practical relevance: the refractoriness to inhibitors of the biological activity of oncogenic protein due to the lack of their molecular target. Thus, LSC hosted in SCN are suited to sustain the long-term maintenance of therapy-resistant minimal residual disease.

## 1. Introduction

Most stem cell studies, and definitely all of the earliest ones, were carried out using hematopoiesis as a model of tissue regeneration. Likewise, hypotheses to describe the tissue environment where normal stem cells are maintained were developed within a hematological context. Schofield organized this issue conceptually in 1978, putting forward the “stem cell niche” (SCN) model [1] to define bone marrow (BM) sites dedicated to the maintenance of hematopoietic stem cells (HSC). The model predicted that this maintenance is achieved by preventing HSC commitment to differentiation. In other words, if HSC proliferate within the SCN environment, they would do without losing stem cell potential, that is, undergoing the so-called “self-renewal,” which is a defining feature of stem cells. The SCN model was supported by experimental findings indicating that HSC and less immature hematopoietic progenitor cells (HPC) are compartmentalized rather than randomly distributed in BM, HSC being located preferentially close to the bone surface and HSC/HPC instead in proximity of the central sinus [2–5]. Hematopoietic cells homing in BM after exogenous transplantation follow a similar compartmentalization pattern [6]. The definition of the relationship between “endosteal SCN,” where HSC are maintained, and “vascular SCN,” where HSC commitment to differentiation and HPC clonal expansion are driven, was completed much later [7, 8]. Hereafter, the acronym SCN is used to indicate SCN where HSC are maintained.

That metabolic factors take part in the regulation of hematopoiesis emerged from studies indicating that low oxygen concentrations in the incubation atmosphere enhance the yield of hematopoietic cultures [9–15]. The issue was deepened in the early 1990s in our laboratory using 1% oxygen to culture murine BM cells. In 1% oxygen, HSC maintenance was enhanced while the overall hematopoietic output was reduced. HPC were indeed suppressed, the more severely the lower their hierarchical level. These studies provided the first mechanistic implementation of the Schofield's SCN model and led to putting forward the hypothesis of a “hypoxic SCN” dedicated to hosting and preserving HSC selectively from HPC [16]. The effects of low oxygen were later confirmed, by us and others, for human hematopoiesis and long-term-repopulating HSC [17–21]. When we addressed directly the role of low oxygen in modulating self-renewal, we found that one replication cycle in cultures incubated in 1% oxygen boosts stem cell potential. Such an effect is lost when cycling is sustained for more than one cycle and does not occur in air or in the presence of interleukin-3 (IL-3). This indicates that HSC self-renewal occurs immediately after HSC rescue from quiescence to cycling, provided this happens in low oxygen, which therefore appears as a crucial factor to spare stem cell potential. Thus, low oxygen maintains HSC in a state where proliferation is allowed, but not commitment to differentiation. The latter is instead typically driven when proliferation is extensively stimulated, such as in the presence of IL-3 [22].

Although tissue areas where stem cell potential is maintained are commonly referred to as “hypoxic SCN,” low-oxygen tensions represent a physiological feature of SCN. Indeed, while the nonphysiological sea-level atmospheric oxygen concentration (20-21%) is considered a physiological standard (“normoxia”) for cell culture incubation, oxygen tensions corresponding to 0.1–5% concentration actually characterize the microenvironment of a number of different tissues. Thus, close to the lower end of this range, oxygen tensions are normoxic for HSC but hypoxic for HPC and the bulk of hematopoietic cell population. This issue is reviewed elsewhere [23–25].

To take advantage of their selective homing in tissue areas at the lowest oxygen tensions, HSC need a complex pattern of metabolic adaptation which is not shared by HPC. It is worth noting here that, in keeping with what summarized above, the term “adaptation” is commonly used with a reverse meaning. The term refers in fact to the capacity of cells to home at the lower oxygen tensions actually found in tissues (“adaptation to hypoxia”), instead of defining the conditions enabling cells to stand the nonphysiologically elevated oxygen concentrations used for cell culture (“adaptation to hyperoxia”) [25]. When it comes to defining the metabolic peculiarity of HSC with respect to HPC, one must specify that this peculiarity does not simply consist of the compatibility with tissue “hypoxia,” as either HSC or HPC exhibit a “hypoxic” metabolic profile [26]. Rather, as mentioned above, HSC, different from HPC, are capable to stand the lowest physiological oxygen tensions (0.1–1%, referred to as very low oxygen) and for extended times. The regulative role of hypoxia-inducible factor-*α* (HIF*α*) signalling on hematopoiesis needs to be considered within this context. HIF1*α* upregulation via transcriptional activation and/or protein stabilization is known as the key driver of cell “adaptation to hypoxia.” However, as its stabilization threshold is 2% oxygen or even higher [27], HIF1*α* alone cannot select HSC from HPC or confer upon HSC all the features enabling their exclusive homing in the very low-oxygen SCN. In other words, HIF1*α* stabilization is necessary but not sufficient condition for HSC maintenance. HIF1*α* activity is indeed required for the maintenance of HSC as well as leukemia stem cells (LSC) of chronic myeloid leukemia (CML) [28, 29]. Thus, while the overall “adaptation to hypoxia” requires HIF1*α* upregulation, enhanced glycolysis and reduced mitochondrial/respiratory activity, only a small minority of hypoxia-adapted cells is capable to stand the very low-oxygen tensions typical of SCN [30]. The characterization of this cell subset is of course of great interest, especially in oncological settings.

The use of 0.1% oxygen in the incubation atmosphere of leukemia cell populations led us to detecting a crucial feature of the SCN-adapted LSC subset [31, 32]. In an environment where all cells are subjected to HIF1*α*-driven metabolic adaptations (see above), we found that only a minority of CML or murine erythroleukemia (MEL) cells persists throughout extended incubation times. In these cells, while stem cell potential is maintained, the oncogenic protein responsible for disease is lost (“oncogene suppression”) [33, 34]. In the case of CML, this loss was found to occur when the shortage of glucose complicates that of oxygen [35]. This points to the existence of a leukemia cell subset combining the adaptation to energy shortage with the suppression of oncogenic signals. Such a combination is likely to be a key factor enabling this cell subset to survive within the selective SCN environment. A crucial by-product of oncogene suppression in CML is that SCN-adapted cells are refractory to the tyrosine kinase inhibitors (TKi) which target the constitutive enzymatic activity of the BCR/Abl oncogenic protein responsible for disease [33, 35], in keeping with findings obtained in other laboratories [36–38]. Refractoriness to therapy due to the fact that its molecular target is suppressed in some surviving cells, LSC in particular, possibly represents the most straightforward explanation of the long-term persistence of therapy-resistant minimal residual disease (MRD) of CML [31, 32]. The data reported in this paper indicated that oncogenic proteins and signals are suppressed in several different leukemias incubated at very low-oxygen concentration. The relevance of this phenomenon as a general aspect of leukemia cell adaptation to severe energy restriction is discussed.

## 2. Materials and Methods

### 2.1. Cells and Culture Conditions

K562 and KCL22 (human CML), NB4 (human acute promyelocytic leukemia), Kasumi-1 (human acute myeloid leukemia), and MEL cell lines were cultured in RPMI 1640 (containing 2 g/l of D-glucose) supplemented with 10% foetal bovine serum, 50 units/ml penicillin, 50 mg/ml streptomycin, and 2 mM/l-glutamine (all from EuroClone, Paignton, UK). Exponentially growing cells were plated at 3 × 10^5^ or 3 × 10^4^ cells/ml and incubated at 37°C in water-saturated atmosphere containing 0.1% O₂, 94.9% N₂, and 5% CO₂ in an anaerobic workstation (Concept 400, Baker Ruskinn, York Road, or DG250, Don Whitley Scientific, Shipley, Bridgend, UK) or in normoxia (21% O₂ and 5% CO₂) in a conventional cell culture incubator. Cell viability was measured by trypan blue (#F-7378, Sigma-Aldrich, St. Louis, MO, USA) exclusion test.

### 2.2. Protein Extraction and Western Blotting

Cells were lysed in Laemmli buffer and protein concentration was determined by the BCA method (Pierce™ BCA Protein Assay Kit; Thermo Fisher Scientific, Waltham, MA, USA). Extracted proteins were subjected to SDS-PAGE and transferred onto polyvinylidene fluoride membranes (Merck-Millipore, Billerica, MA, USA) by electroblotting. Membranes were blocked in a 1 : 1 dilution of Odyssey blocking buffer (OBB; LI-COR® Biosciences, Lincoln, NE, USA) with PBS and then incubated at 4°C overnight with primary antibody in a 1 : 1 dilution of OBB with PBS-0.1% Tween 20 (T-PBS). Primary antibodies used were rabbit polyclonal anti-pCRKL (#3181) from Cell Signaling Technology (Danvers, MA, USA); rabbit polyclonal anti-c-Abl (sc-131), anti-erythropoietin-receptor (EPo-R; sc-697) and anti-ERK1 (sc-93), mouse monoclonal anti-RAR*α* (sc-515796) and anti-tubulin (sc-32393), and goat polyclonal anti-ETO (sc-9737) and anti-GAPDH (sc-20357) from Santa Cruz Biotechnology (Santa Cruz, CA, USA); rabbit polyclonal anti-histone 4 (H4; #07-108) from Merck-Millipore; goat anti-R-MuLV gp70 antiserum, recognizing gp55 in MEL cells, kindly provided by Dr. Sandra Ruscetti (Laboratory of Cancer Prevention, National Cancer Institute, Frederick, MD, U.S.A.); and rabbit anti-ARD1, produced in Dr. Nathalie Mazure's laboratory. After washing with T-PBS, membranes were incubated for 1 h at room temperature in OBB 1 : 1 with PBS containing IRDye800CW (1 : 20000)- or IRDye680 (1 : 30000)-conjugated secondary antibody (LI-COR). Antibody-coated protein bands were visualized by Odyssey Infrared Imaging System Densitometry (LI-COR Biosciences, Lincoln, NE, USA), as previously reported [39].

### 2.3. Measurement of Glucose Concentration in Culture Medium

Medium samples were harvested at the indicated times and stored frozen at −20°C until analysis was performed by the glucose hexokinase method using the ADVIA 2400 Chemistry System (Siemens, Camberley, Surrey, UK).

### 2.4. Statistical Analysis

Statistical analyses were performed using Student's *t*-test and GraphPad Prism software. A *p* value less than 0.05 was considered statistically significant.

## 3. Results

**Figure 1:**
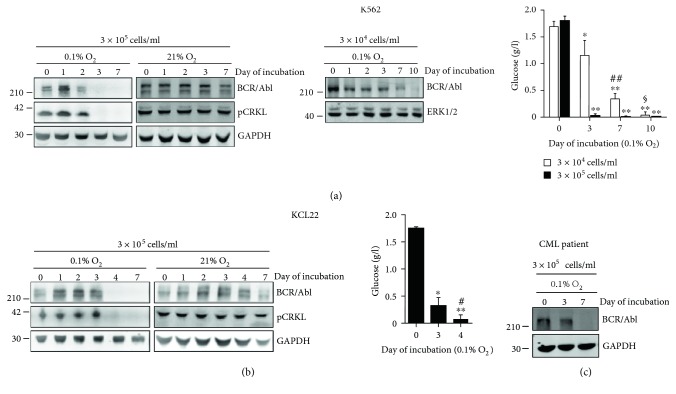
Suppression of BCR/Abl protein in CML cells undergoing “adaptation to hypoxia.” K562 cells (a), KCL22 cells (b), or blast-crisis primary cells (c) were plated at the indicated time-zero cell concentrations and incubated in atmosphere at 0.1% or 21% O_2_. Cell lysates obtained at the indicated incubation times were immunoblotted using anti-c-Abl (detecting BCR/Abl) or anti-phospho-CRKL antibodies or, as loading equalization control, anti-GAPDH or anti-ERK1/2 antibodies; migration of molecular weight markers is indicated on the left (kDa). One out of three independent experiments with similar outcome is shown. Glucose concentration in the medium of cultures incubated at 0.1% O_2_ was measured at the indicated incubation times as described in Materials and Methods. Values are means ± SD of data from 3 independent experiments; ^∗^*p* ≤ 0.05 and ^∗∗^*p* ≤ 0.01 versus time 0; ^#^*p* ≤ 0.05 and ^##^*p* ≤ 0.01 versus day 3; ^§^*p* ≤ 0.01 versus day 7.

The incubation of CML cell lines in atmosphere at 0.1% O_2_ was paralleled by a time-dependent suppression of BCR/Abl protein, which did not occur in cells incubated at 21% O_2_ (Figures 1(a) and 1(b)), in keeping with what was previously shown [33, 35, 40]. These findings are confirmed and extended here to primary CML cells explanted from BM of a patient in blast crisis (Figure 1(c)). Figures 1(a) and 1(b) also show that incubation in low oxygen led to a decrease in the phosphorylated form of CRLK, a major BCR/Abl downstream substrate used as read-out of BCR/Abl activity. Thus, the suppression of BCR/Abl protein abolished, as expected, its tyrosine kinase activity. The kinetics of BCR/Abl suppression varied depending on the cell line analysed. Indeed, in K562 cells, BCR/Abl protein level was undetectable starting from day 3 of incubation in low oxygen (Figure 1(a), blot on the left), while in KCL22 cells, BCR/Abl protein suppression occurred at day 4 (Figure 1(b)).

Previous studies carried out in our laboratory revealed a close relationship between suppression of BCR/Abl protein in CML cells and glucose exhaustion from culture medium [35]. These findings are confirmed and extended here by measuring the time course of glucose concentration in cultures of KCL22 cells and in K562 cells plated at different concentrations (Figures 1(a) and 1(b), graphs). In the experiments carried out with KCL22 cells, glucose got exhausted between day 3 and day 4, when BCR/Abl protein expression became undetectable (Figure 1(b)). In K562 cell cultures where 3 × 10^5^ cells/ml were plated at time zero, glucose was already exhausted on day 3, when BCR/Abl protein expression became undetectable (Figure 1(a), blot on the left). In K562 cell cultures established with 3 × 10^4^ cells/ml, glucose was still relatively high on day 7 and exhausted on day 10, findings perfectly in keeping with those relative to BCR/Abl protein expression (Figure 1(a), blot on the right). These data confirmed the relationship between glucose consumption and BCR/Abl protein suppression.

**Figure 2:**
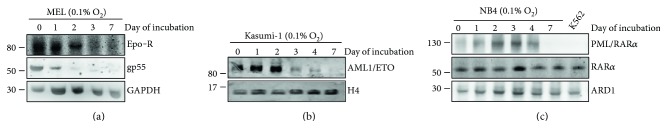
Suppression of oncogenic proteins driving non-CML blood neoplasias in the course of cell “adaptation to hypoxia.” MEL (a), Kasumi-1 (b), or NB4 (c) cells were incubated in atmosphere at 0.1% O_2_ and lysed at the indicated times, and total cell lysates were subjected to immunoblotting with the indicated antibodies. GAPDH, H4, or ARD1 were detected to verify loading equalization. Migration of molecular weight markers is indicated on the left (kDa). For each cell population, one out of three independent experiments with similar outcome is shown.

The effects of incubation at 0.1% O_2_ of a number of non-CML leukemia cell lines are shown in Figure 2. MEL and Kasumi-1 cells underwent a time-dependent suppression of the oncogenic protein(s) driving the disease (Figures 2(a) and 2(b)), in keeping with previous observations [34, 41]. These findings are extended here to NB4 cells (Figure 2(c)). It is to note in particular that in MEL cells, either the gp55 protein of Friend's virus or the EPo-R, both contributing to oncogenic signalling in these cells, was suppressed. In Kasumi-1 cells, the oncogenic driver AML1/ETO protein behaved likewise. In NB4 cells, incubation in low oxygen led to the suppression of the oncogenic driver PML/RAR*α*, but not the normal RAR*α* protein; a lysate of K562 CML cells was added to the electrophoretic run as negative control.

## 4. Discussion

This paper shows that incubation at very low-oxygen tensions determines the suppression of driver oncogenic proteins and signals in a number of leukemia cell populations, leading to hypothesizing that this suppression is a widespread phenomenon occurring in different types of cancers, including solid neoplasias. That the triggering of suppression mechanism is related to the onset of severe energy restriction rather than simply to “adaptation to hypoxia” is supported by the fact that, in all types of leukemia tested, oncogene suppression occurred only after 3-4 days of incubation in very low oxygen. Consequently, the phenomenon cannot be simply (or directly) ascribed to HIF1*α* activation, which is driven within minutes of cell challenged with oxygen shortage. Based on the previous finding that BCR/Abl suppression in low oxygen parallels the onset of glucose shortage [35] and on the data reported here, what we call “severe energy restriction” seems actually to consist of glucose exhaustion from culture medium.

The time-dependent suppression of oncogenic proteins we found as a widespread response of leukemia cells to incubation in low oxygen needs to be discussed within the issue of the general effects of “hypoxia” on protein expression [42]. Indeed, the ATP demand for protein synthesis under “hypoxia” has been estimated to drop to about 7% of that in “normoxia” [43]. This drop occurs initially at the level of translation and later extends to transcription level [44]. Despite such a general decrease in protein synthesis, mRNA translation continues of course for factors important for adaptation to “hypoxia,” such as HIF-*α* and HIF-*β*, vascular endothelial growth factor, and platelet-derived growth factor [45]. On the other hand, we found that, in CML cells, a number of not directly hypoxia-related proteins, such as tubulin for example, are not affected by energy shortage following either oxygen or glucose deprivation [40]. However, the relationship of downregulation of protein expression to oxygen and glucose shortage remains to be deepened.

**Figure 3:**
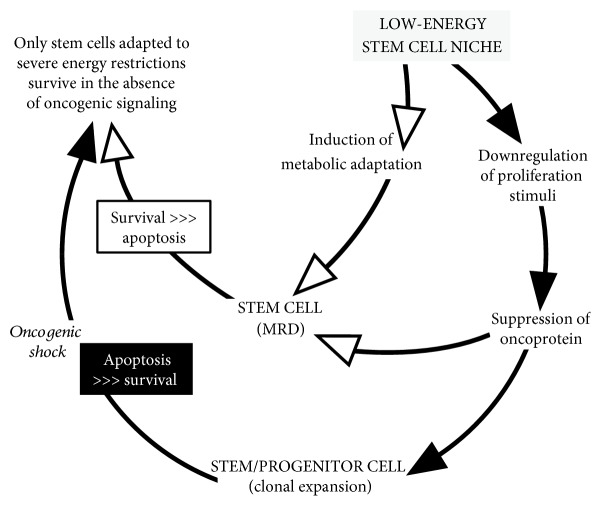
Metabolic adaptation lets stem cell escape oncogene addiction and oncogenic shock. Suppression of oncogenic signalling is necessary to prevent stimuli driving commitment to clonal expansion and differentiation from antagonizing the long-term maintenance of stem cell properties in the SCN. Oncogene suppression puts under stress (black arrowheads/box) stem/progenitor cells committed to clonal expansion and differentiation, which are oncogene-addicted. Thus, in the SCN, these cells would be subjected to prevalent proapoptotic stimuli and undergo the “oncogenic shock.” On the contrary, stem cells which metabolically adapt to SCN environment become independent of oncogene signalling (lose oncogene addiction) and escape oncogenic shock (white arrowheads/box), ensuring MRD maintenance.

As briefly mentioned in the Introduction, oncogene suppression under severe energy restriction most likely mirrors the fact that this condition is incompatible with sustained oncogenic signalling which drives commitment to clonal expansion and differentiation. The experiments reported in this paper did not address directly the regulation of LSC maintenance in the function of the expression of driver oncogenes. However, previous data obtained for some of the cell lines used here indicate that, under conditions driving oncogene suppression, cell growth is markedly reduced while stem cell potential is not suppressed [33–35]. In this respect, it is worth pointing out that stabilized leukemia cell lines are highly heterogeneous populations which comprise a full spectrum of different functional phenotypes and include cycling or quiescent cell subsets endowed with stem cell potential [34]. Based on these premises, one can propose that LSC are capable in principle to survive in the absence of oncogenic signalling and that metabolic pressure in the low-energy SCN exploits this capacity and actually selects LSC which have lost oncogene addiction and are capable to stand oncogene suppression (Figure 3). On the contrary, in LSC which do not undergo metabolic adaptation, oncogene addiction is maintained, so that oncogene suppression would result in the induction of apoptosis, the so-called “oncogenic shock.” In other words, maintained oncogenic stimulation (enforcing commitment to differentiation and clonal expansion) makes LSC unfit to home in SCN, where they would suffer of the prevalence of proapoptosis over prosurvival stimuli upon withdrawal of oncogenic signalling [46–48].

The loss of oncogene addiction within the SCN implies a de facto reversion of LSC to the normal HSC phenotype, as long as they remain under conditions where the balance within the regulation of LSC compartment is in favour of the maintenance of stem cell potential rather than of commitment to differentiation [48]. Revertant LSC are likely to rely on physiological extra-/intra-cellular signals for support of their survival and self-renewal. In other words, the loss of oncogenic signalling in LSC should be considered functionally equivalent to HSC deprivation of cytokine signalling which drives commitment to differentiation and extensive proliferation, such as that of IL3 [22]. However, LSC adapted to the SCN environment maintain their leukemic genetic signature, so that they are capable, when transferred to growth-permissive conditions outside the SCN, to regenerate oncogenic protein-expressing/protein-dependent cells and thereby to rescue drive to clonal expansion and relapse of disease [33, 35, 40]. Thus, oncogene suppression is not a genetically blocked event but a fully reversible phenotypical adaptation, according to the “chiaroscuro stem cell” model proposed by Quesenberry et al. in 2002 to define the relationship between the HSC and HPC phenotypes [49]. This model may be integrated with the model, proposed by Reya and coworkers, defining two alternative scenarios for the generation of cancer stem cells [50], that is, the oncogenic transformation of a normal (self-renewing) stem cell or the staminalization (acquisition of self-renewal) of a normal progenitor cell. Our data seem to indicate that these two scenarios are to be considered complementary rather than alternative and to correspond, respectively, to LSC capable or incapable to adapt to and home in SCN.

A crucial, and of high practical relevance, consequence of oncogene suppression is that SCN-adapted LSC which reverted to a normal HSC phenotype exhibit complete refractoriness to inhibitors of the biological activity of the oncogenic protein, due to the lack of their molecular target [31, 32]. The combination of refractoriness to therapy with long-term persistence in tissues makes SCN-adapted LSC the best candidates to sustain MRD in vivo. As far as CML is concerned, the presence of these LSC is probably the main reason of the failure of BCR/Abl-targeting TKi to eradicate LSC, suppress MRD, and prevent relapse of disease [51]. In this scenario, it is predictable that even next-generation TKi will be equally ineffective, as we already observed in vitro (manuscript submitted). All above urges to investigate on the mechanisms of LSC adaptation to severe oxygen and nutrient shortage in view of the design of therapeutic strategies directed to eradicate, rather than to control, leukemia. However, a drawback preventing a useful application of such strategies is represented by the revertant nature of metabolically adapted LSC, which implies the risk of seriously damaging the HSC pool while trying to eradicate LSC. In this respect, current therapeutic approaches directed to suppress oncogene-addicted leukemia/cancer cells committed to clonal expansion and differentiation while tolerating the maintenance of LSC self-renewal at the subclinical level may turn out to represent a safer scenario.
